# Mapping the Tail Fiber as the Receptor Binding Protein Responsible for Differential Host Specificity of *Pseudomonas aeruginosa* Bacteriophages PaP1 and JG004

**DOI:** 10.1371/journal.pone.0068562

**Published:** 2013-07-09

**Authors:** Shuai Le, Xuesong He, Yinling Tan, Guangtao Huang, Lin Zhang, Renate Lux, Wenyuan Shi, Fuquan Hu

**Affiliations:** 1 Department of Microbiology, Third Military Medical University, Chongqing, China; 2 School of Dentistry, University of California Los Angeles, Los Angeles, California, United States of America; Centro Nacional de Biotecnologia - CSIC, Spain

## Abstract

The first step in bacteriophage infection is recognition and binding to the host receptor, which is mediated by the phage receptor binding protein (RBP). Different RBPs can lead to differential host specificity. In many bacteriophages, such as *Escherichia coli* and *Lactococcal* phages, RBPs have been identified as the tail fiber or protruding baseplate proteins. However, the tail fiber-dependent host specificity in *Pseudomonas aeruginosa* phages has not been well studied. This study aimed to identify and investigate the binding specificity of the RBP of *P. aeruginosa* phages PaP1 and JG004. These two phages share high DNA sequence homology but exhibit different host specificities. A spontaneous mutant phage was isolated and exhibited broader host range compared with the parental phage JG004. Sequencing of its putative tail fiber and baseplate region indicated a single point mutation in ORF84 (a putative tail fiber gene), which resulted in the replacement of a positively charged lysine (K) by an uncharged asparagine (N). We further demonstrated that the replacement of the tail fiber gene (ORF69) of PaP1 with the corresponding gene from phage JG004 resulted in a recombinant phage that displayed altered host specificity. Our study revealed the tail fiber-dependent host specificity in *P. aeruginosa* phages and provided an effective tool for its alteration. These contributions may have potential value in phage therapy.

## Introduction

*Pseudomonas aeruginosa* is an opportunistic pathogen that can cause a wide range of acute and chronic infections in cystic fibrosis, cancer, and burn patients, as well as in immunocompromised individuals [1–6]. *P. aeruginosa* remains a clinically important bacterial pathogen mainly because of its ability to develop resistance to antibiotics [7,8]. To fight this bacterium, alternative therapeutic strategies are explored, such as developing vaccines against *P. aeruginosa* and suppressing expression of virulence factor by inhibiting the quorum-sensing signals [9,10]. One of the promising alternative approaches to conventional antibiotics treatment is phage therapy [9,11–13]. The success of this treatment greatly depends on the host specificity of the phage. Specificity is often determined by the interaction between a phage receptor-binding protein (RBP) and a specific receptor on the surface of the host cell [14–19].

The first step in phage infection is adsorption of the phage to the host cell [20]. Recently, a single-virus tracking experiment using fluorescently labeled phages accurately described the initial process [21]. Phages diffuse randomly until they encounter a bacterial cell. Then, the phages will continue to diffuse on the cell surface until they bind to a receptor that initiates the infection process. Otherwise, phages fall off from the cell surface and continue their free motion. The binding process usually has two steps: reversible and irreversible binding. For example, in T4-like phages, the attachments of long tail fibers to specific receptors are reversible [22]. After attachment by long fibers, the baseplate changes its shape, and six short fibers extend and irreversibly bind to the lipopolysaccharides (LPS) core. This process generates a signal transmitted to the phage head and triggers DNA release. However, the baseplate of TP901-1-like phages is in a conformation ready for host binding, and thus, does not rely on conformational change [23]. The detailed mechanisms vary in different phages, but they are all determined by both the receptor on the cell surface and the RBP in the phage. In *Escherichia coli* and lactic acid bacteriophages, such as p2, T4 and Lambda, these RBPs have been identified as tail fibers or protruding baseplate proteins that have been well studied [17,24]. In phage Lambda, the C-terminal portion of the tail fiber allows phage to adsorb to the surface of *E. coli* K-12 by interacting with the outer membrane receptor *LamB* [24]. *E. coli* phages T5 and BF23 use the tail shaft, which is located above the straight tail fiber, as RBPs [16]. The RBPs of temperate *Lactococcal* bacteriophages TP901-1 and Tuc2009 are mapped and characterized as lower baseplate proteins [23,25]. Interestingly, the host range of lytic phage phi92 is not limited to *E. coli* but also includes *Salmonella* . By analyzing the structure of phage phi92, Schwarzer et al. indicated the four different types of tail fibers and/or tail spikes, which allow the phage to attach to encapsulated and nonencapsulated bacteria [19]. A total of 53 *Pseudomonas* phages have been fully sequenced and submitted to the NCBI database. However, the RBPs of these phages are not identified, and the knowledge of phage–host interactions is still very limited.

In our previous studies, we isolated and sequenced three *P. aeruginosa* phages [26–28], including lytic *P. aeruginosa* phage PaP1, which is a Myoviridae phage isolated from sewage. Sequence analysis showed that PaP1 (GenBank: HQ832595.1) [26] shares high sequence similarity with another *P. aeruginosa* phage JG004 (GenBank: GU988610) [29], including the region that encodes putative tail and baseplate structural proteins. The current study aimed to identify the RBPs of these two *P. aeruginosa* phages and to investigate their RBP-dependent host specificity.

## Results

### Differential host specificity of phages PaP1 and JG004

**Figure 1:**
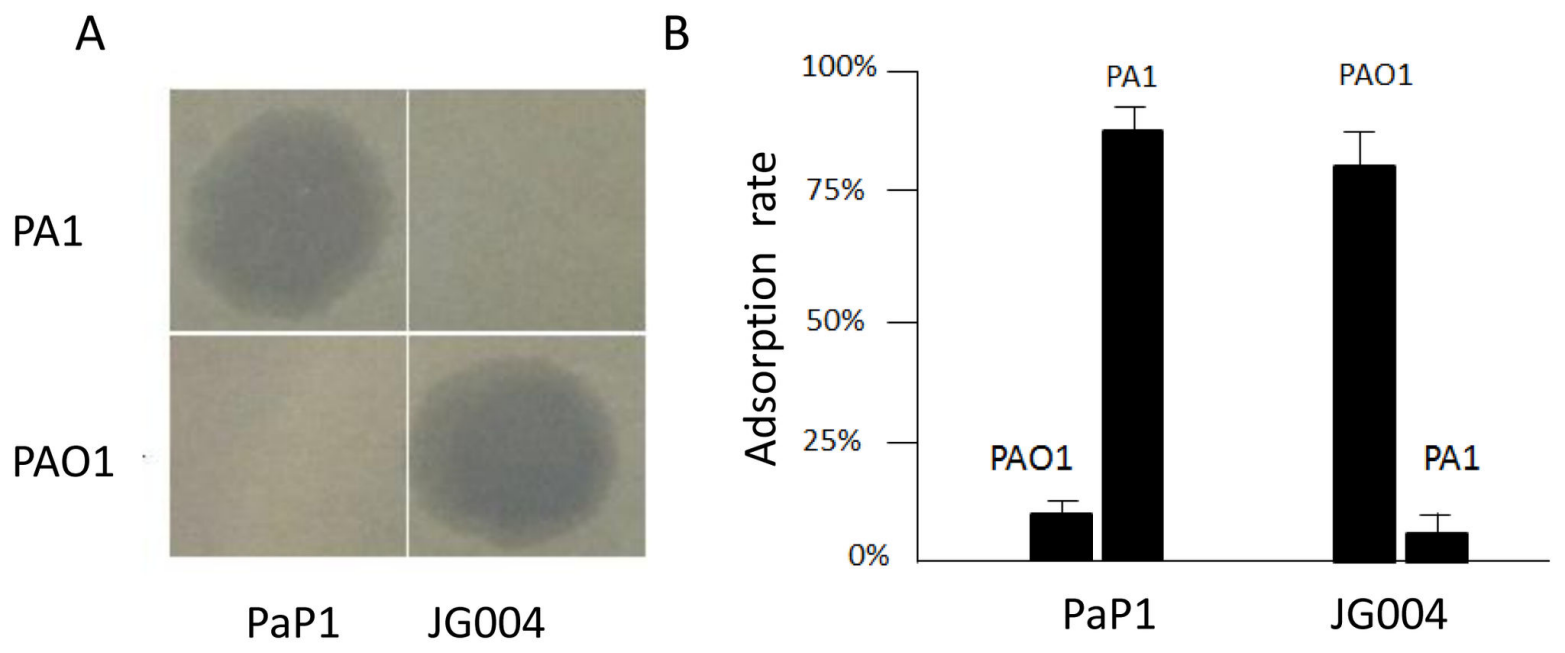
Phages PaP1 and JG004 show different host specificities. **A**: Spot assays for testing host specificity of phages PaP1 and JG004. **B**: Adsorption assay of bacteriophages PaP1 and JG004 to *P. aeruginosa* PA1 and PAO1. Percent adsorption of the phage was calculated as [(initial titer − residual titer) / initial titer] × 100%.

PaP1 was initially isolated using *P. aeruginosa* PA1 as host strain. JG004 was shown to infect PAO1 strain. Comparative sequence analysis indicated a striking sequence similarity between these two phages [26,29]. However, our spot assay showed that PaP1 only infected PA1, whereas JG004 specifically induced plaque formation on PAO1. No cross-infection was observed for either phage (Figure 1A). An adsorption assay was performed to test whether the inability of PaP1 to infect PAO1 was due to its defect in the initial adsorption or bacteria-encoded phage resistance mechanisms affecting the later steps during phage infection process. As shown in Figure 1B, PaP1 was unable to adsorb to PAO1 and JG004 failed to bind to PA1. Our data suggested that PaP1 and JG004 may encode different RBPs that are responsible for differential host specificity.

### Isolation and characterization of spontaneous mutants of phage JG004 that are capable of infecting PA1

**Figure 2:**
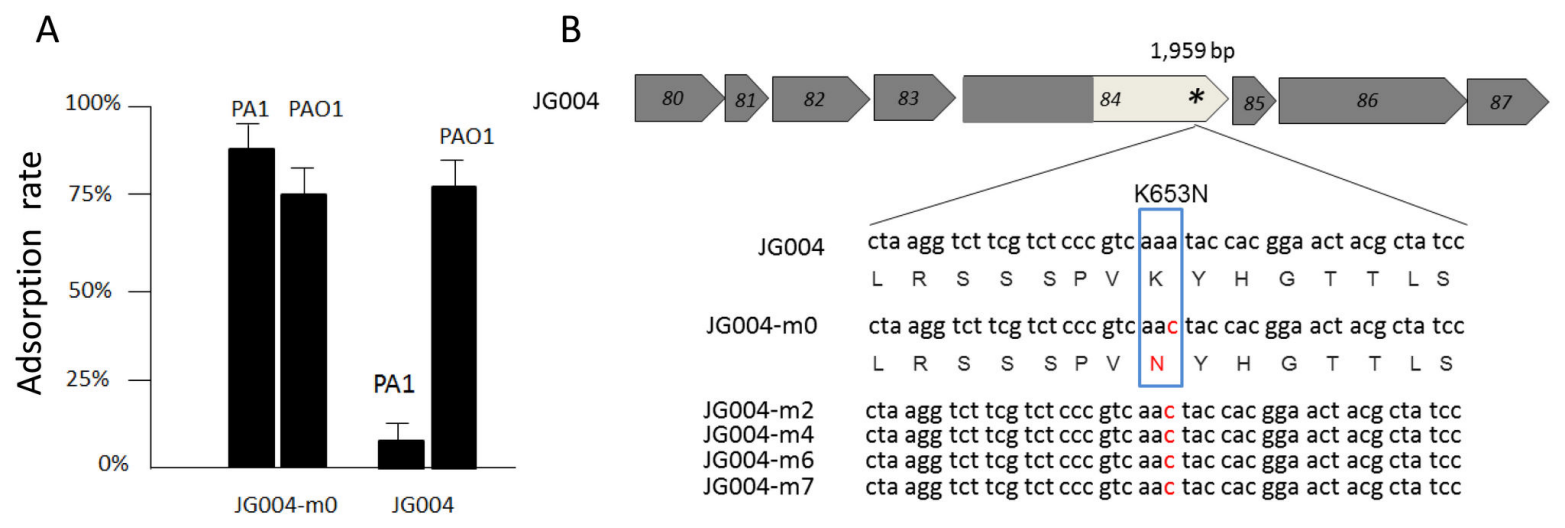
Characterization of spontaneous mutant phages. **A**: Adsorption assay of bacteriophages JG004-m0 and JG004 to *P. aeruginosa* PA1 and PAO1. **B**: Gene organization of the region encoding tail proteins in JG004. The star in ORF84 indicates an A1959C mutation in ORF84 (2058 bp) in mutant phage JG004-m0. The alignment of the DNA and amino acid sequences of ORF84 between phage JG004 and five mutant phages indicates that the adenine (A) residue in phage JG004 changed to cytosine (C) in the mutant phages (shown in red).

To identify the phage components responsible for host specificity, a genetic approach was used to isolate spontaneous mutants of phage JG004 that acquired the ability to infect *P. aeruginosa* strain PA1. Our result showed that these phage mutants could be consistently obtained with a frequency of approximately 10^−8^. Five spontaneous phage mutants (JG004-m0, m2, m4, m6, and m7) were isolated, and JG004-m0 was selected for further analysis. Interestingly, the adsorption assay showed an equally efficient binding of JG004-m0 to both PA1 and PAO1 strains (Figure 2A).

Given that the phage tail fiber or baseplate is often responsible for the adsorption specificity of phage [18,19], we suspected that the mutation of JG004-m0 may be located within the genetic loci encoding those phage structures. Eleven pairs of primers (Table S1) were designed to amplify the whole baseplate and tail fiber region (ORF76 to ORF86) of mutant phage JG004-m0 and parental phage JG004.

Compared with the JG004 sequence, a single point mutation was detected in JG004-m0 within the ORF84. ORF84 is an open reading frame predicted to encode a putative tail fiber protein composed of 685 amino acids (aa) (Figure 2B). The adenine (A) residue at nucleotide position 1,959 in ORF84 [2,058 base pairs (bp)] in the wild-type phage JG004 was changed to cytosine (C) in the mutant phage JG004-m0. This change resulted in the replacement of a positively charged lysine (K) at the amino acid position 653 in the phage tail fiber, with an uncharged asparagine (N) in the mutant phage JG004-m0.

To further test if the same single point mutation can be detected in other isolated mutant phages, four more mutant JG004 phages (JG004-m2, JG004-m4, JG004-m6, and JG004-m7), which are capable of binding to both PA1 and PAO1 (Figure S1), were subjected to comparative sequence analysis. The result indicated that all four mutant phages carried the same point mutation at the C-terminus of ORF84 (Figure 2B).

Interestingly, the spontaneous PaP1 mutant phage, which is capable of infecting PAO1, could not be obtained using a similar genetic approach.

### The C-terminus of the tail fiber gene of PaP1 and JG004 is highly diversified

**Figure 3:**
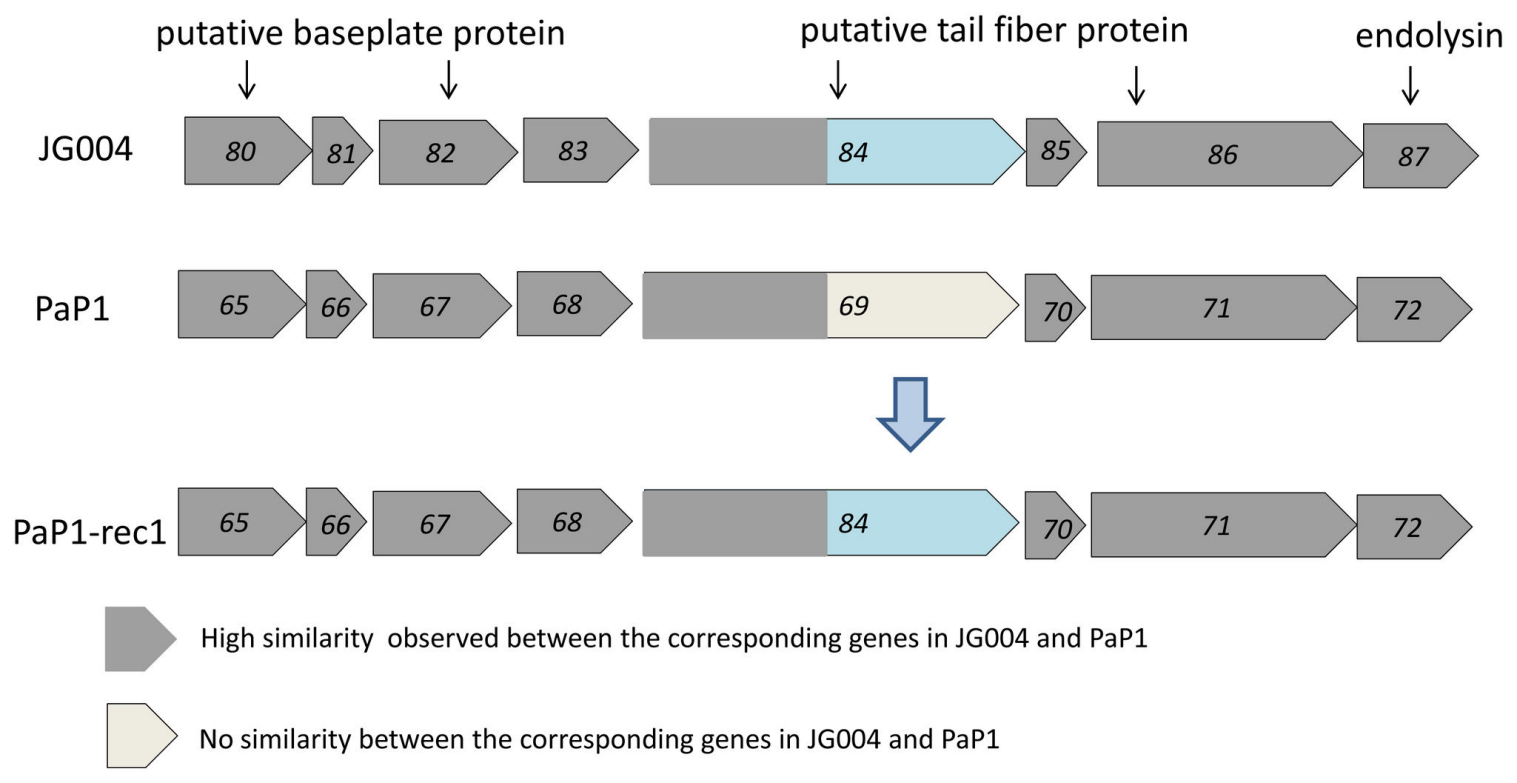
Comparison of genetic loci encoding putative tail fiber and baseplate between JG004 and PaP1. Dark gray indicates high similarity between corresponding genes in JG004 and PaP1. Light gray indicates the absence of similarity between the corresponding region in JG004 and PaP1.

A point mutation within a putative tail fiber-encoding gene (ORF84) resulted in an altered host range, suggesting the involvement of the tail fiber in the host specificity of phage JG004. The differential host specificity displayed by PaP1 and JG004 also indicated that although they have overall high sequence similarity, their tail fiber and baseplate region may be diverse enough to confer the observed host specificity. Bioinformatics analysis was applied to compare the genetic loci encoding putative tail fiber and baseplate in phages PaP1 and JG004 (Figure 3). Results showed that most of the putative tail fiber and baseplate encoding genes between PaP1 and JG004 shared high similarity (99%). However, the two predicted tail fiber genes ORF69 and ORF84 of PaP1 and JG004, respectively, displayed high levels of sequence diversity. Interestingly, ORF69 had 2,013 bp (670 aa), whereas ORF84 of JG004 contained 2058 bp (685 aa). Furthermore, the N-terminus 969 bp between ORF84 from JG004 and ORF69 from PaP1 shared a relative similarity of 88%. Genes flanking ORF84 in JG004 were both 99% identical to the corresponding region of PaP1. However, no similarity was observed in the C-terminal regions.

### Generation and characterization of chimeric phage with altered host specificity

**Figure 4:**
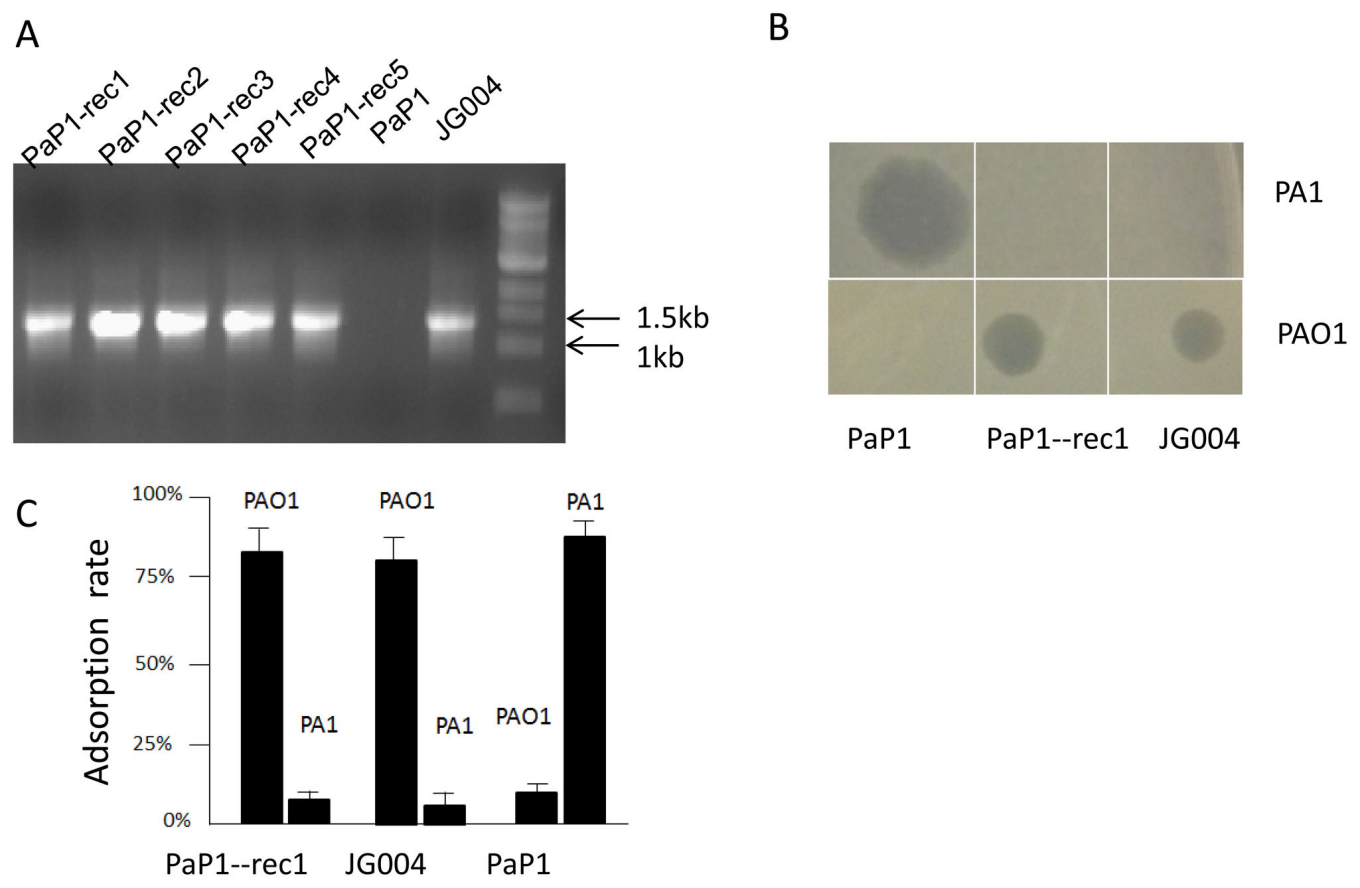
Characterization of recombinant phages. **A**: PCR assay for confirmation of recombinant phages. **B**: Spot assay for testing the ability of PaP1, PaP1-rec1, and JG004 to infect PAO1 and PA1. **C**: Adsorption assay for testing the specificity of JG004, PaP1, and recombinant PaP1-rec1 in binding to *P. aeruginosa* strains PA1 and PAO1.

Comparative sequence analysis suggested that similar to ORF84 which encodes a putative RBP in JG004, the ORF69 in PaP1 is likely to encode an RBP, which could determine its host specificity. We proposed that by replacing the ORF69 in phage PaP1 with the ORF84 from phage JG004, we should be able to construct a chimeric PaP1 phage that is able to infect PAO1 instead of PA1 (Figure 3). The high similarity (99% identity) between the sequences flanking ORF84 in JG004 and ORF69 in PaP1 allowed us to construct recombinant PaP1 phage with its ORF69 replaced by ORF84 from JG004 by homologous recombination. The resulting recombinant PaP1 phages, namely, PaP1-rec1, PaP1-rec2, PaP1-rec3, PaP1-rec4 and PaP1-rec5, were confirmed by a diagnostic polymerase chain reaction (PCR) using JG004-specific primers (JG004-84-specific U/D). These primers amplified a fragment located within the C-terminal of ORF84. The expected PCR product sizes were generated from five recombinant phages and JG004, but not from PaP1 (Figure 4A). The sequencing analysis of these PCR products further confirmed that the ORF69 of phage PaP1 was successfully replaced by ORF84 from JG004. Meanwhile, when recombinant PaP1 phage DNA was used as template, PaP1-specific primers (JG-P1U/D) were able to amplify the PCR products of the same size and sequence with parental PaP1 (data not shown). This result indicated that the obtained phages were recombinant and not a contamination of other unrelated phages.

Spot assay showed that the recombinant phage PaP1-rec1 was able to induce plaque formation on PAO1 strain but not on PA1, which is the original host strain for wild-type PaP1 (Figure 4B). To test if the inability of PaP1-rec1 to infect PA1 was due to a defect in phage adsorption as a result of RBP replacement, an adsorption assay was performed. The result (Figure 4C) showed that PaP1-rec1 displayed a strong binding to PAO1, but a very limited adsorption to PA1. The observed adsorption characteristic of the recombinant PaP1 phage was very similar to JG004 but different from its parental phage PaP1. These data strongly indicated that replacement of the tail fiber gene changed the host specificity of phage PaP1.

## Discussion

Multidrug-resistant opportunistic pathogen *P. aeruginosa* poses a major threat to immunocompromised patients [2–5]. Given the paucity of novel antibiotics, phage therapy is proposed as a potential alternative treatment for *P. aeruginosa* infection. However, knowledge on the mechanisms of phage infectivity and bacterial resistance in *P. aeruginosa* and associated phage remains lacking. In particular, the initial irreversible binding of *P. aeruginosa* phage to its host strain, which is the key step in the specific interaction between host receptor and phage RBP, is poorly understood. In a previous study, we isolated *P. aeruginosa* phage PaP1 from sewage. Sequence analysis showed its high similarity with the phage JG004 that was originally isolated from a different *P. aeruginosa* host strain PAO1. In this study, we further identified and characterized the PaP1 and JG004 phage genes involved in host recognition.

JG004 and PaP1 shared overall high sequence similarity; however, they indicated distinct host specificities. Detailed comparative sequence analysis showed striking sequence diversity between the C-terminus of the putative tail fiber-encoding ORF84 in JG004 and ORF69 in PaP1. This result indicated that the C-terminal domains of the encoded tail fibers of the two phages were likely determinants of the host specificity (Figure 3). This finding agreed well with recent studies where the RBP of *Lactococcus lactis* phage sk1, TP901-1, and bIL170 showed a conserved N-terminus and a variable C-terminus [15,30]. Similarly, in bacteriophage Lambda, the C-terminal domain of the tail fiber protein is responsible for binding to the receptor protein LamB [24]. Our data suggested that the conserved N-terminal portion of the tail fiber in JG004 and PaP1 may be involved in a strong interaction with other phage tail proteins. The variable C-terminal regions are responsible for recognizing the host receptor.

Currently, tailed bacteriophages are divided into three families based on their tail morphologies. These families are Podoviridae, Myoviridae, and Siphoviridae, which have short, long contractile, and long non-contractile tails, respectively [31]. The presence of a baseplate and tail fibers is generally a good indicator of phages interacting only with saccharide components, such as Podoviridae and Myoviridae families. For example, the C-terminal of phage TP901-1 tail fiber harbors a saccharide binding site that only interacts with saccharide components [23,30]. A simple tail tip generally indicates that the irreversible adsorption step involves a protein receptor. For example, *Bacillus subtilis* phage SPP1 binds to the membrane protein YueB[32]. . In this study, JG004 and PaP1 both belong to the Myoviridae family, and transposon mutagenesis result shows that O antigen biosynthesis genes are required for phage JG004 infection [29]. Thus, we propose that the initial step of JG004 and PaP1 infection is the adsorption of long tail fibers to the O polysaccharide at the surface of the host.

Interestingly, an A1959C mutation in ORF84 (2058 bp) enabled mutant phage JG004-m0 to infect both PA1 and PAO1 (Figure 2). The mutation is located within the variable C-terminus of the tail fiber-encoding gene (ORF84) in JG004 and indicates that the end of the tail fiber is critical for binding to the receptor. Our result was corroborated by the observation that phages can drastically change their binding affinity to a specific host receptor or even expand their host range by one or a few amino acid substitutions. Similarly, Rainey et al. isolated an extended host range mutant subgroup B avian sarcoma leukosis virus, which had two adjacent amino acid substitutions in their envelope glycoprotein SU (gp85) [33]. In addition, a mutation at the carboxyl end of the 553 amino acid tail fiber protein in *E. coli* phage T7 changed the host specificity to another *E. coli* strain [34]. In *Enterococcus faecalis* phage ΦEF24C, a point mutation in the putative fiber protein ORF31 resulted in a mutant phage ΦEF24C-P2 with drastically increased adsorption ability and infectivity [35]. Bacteria and their associated phages are often engaged in antagonistic coevolution, reciprocal evolution of bacterial resistance, and phage infectivity. During this ‘arms race,’ bacteria with mutated receptors escape phage infection. Meanwhile, mutation in the RBPs is an effective strategy for phages to survive the competition [36–38]. The aforementioned mutation in the mutant phage JG004-m0 resulted in the replacement of a positively charged lysine (K) to an uncharged asparagine (N) from the nucleotide substitution at position 47,213 of the phage genome. Studies in coliphage of the T2 family showed that the majority of substitutions causing changes in receptor specificities of phages were tyrosine, tryptophan, and asparagines. These amino acids could possess structural and functional properties that contribute to the generation of a new binding site [39]. Recently, several RBPs, including the tail fiber of phages T4 and T7, as well as the baseplate of phage p2 and TP901-1 [22,23,40,41], have been successfully crystallized. The detailed structural analysis of these proteins and the co-crystallization of tail fiber and its receptor, such as bacterial LPS fragments, would help in more accurately identifying the specific residues in the tail fiber responsible for receptor binding.

Phage RBPs are potential targets for phage genetic modification to artificially control phage specificity, which may contribute to practical applications of phage therapy. By replacing ORF69 in *P. aeruginosa* phage PaP1 with the corresponding gene ORF84 from JG004, we successfully created a chimeric phage PaP1-rec1 that acquired the host range of phage JG004. The result demonstrated the involvement of the identified tail fiber genes ORF69 and ORF84 in host recognition by phage PaP1 and JG004, respectively. The same approach has been used to genetically modify the RBPs of *E. coli* phage T2 and *Streptococcus* *thermophiles* bacteriophage DT1 to alter their host specificity [14,17]. Differential recombination rates have been observed when constructing chimeric phages, ranging from 10^−4^ to 10^−10^, depending on the different bacteria and their associated phages tested [17,42]. We obtained a moderate recombination frequency of approximately 10^−8^ to 10^−9^, which could be the result of a similar recombination event similar to that in *S* *. thermophiles* cells upon infection [14]. Our results demonstrated that the tail fibers of *P. aeruginosa* phages were involved in host recognition, and that they can be replaced with tail fibers from related phages to acquire different host ranges. A more detailed understanding of the structural and functional interaction between tail fiber and host receptor will enable more precise amino acid substitution and gene modification of tail fibers to expand phage host ranges, which could have potential value in phage therapy.

## Materials and Methods

### Bacterial strains, phage, plasmids, and culture conditions

Table 1 lists the bacterial strains, phages, and plasmids used in this study. *P. aeruginosa* strains were grown in LB medium (5 g yeast extract, 10 g tryptone, 10 g NaCl per liter) [26]. Gentamicin was added at 60 and 10 µg/ml for *P. aeruginosa* and *E. coli*, respectively. All the strains and phages were cultured at 37 °C with aeration.

**Table 1:** Bacterial strains, plasmids, and phages.

**Bacterial strain, plasmid, or phage**	**Relevant feature(s)^a^**	**Reference or source**
*Pseudomonas aeruginosa*		
Strains		
PA1	Host strain for phage PaP1	[26]
PAO1	Host strain for phage JG004	[29]
PA1-p84	PA1+pUCP-84	This study

Phages		
PaP1	Lytic phage	[26]
JG004	Lytic phage	[29]
JG004-m0/m2/m4/m6/m7	Spontaneous mutant phages	This study
PaP1-rec1/2/3/4/5	Recombinant phages	This study

Plasmid		
pUCP24	4.0 kb; coloring vector, Gm^r^	[44]
pUCP-84	8.5 kb; p UCP24::JG004 (nt 44,484–49,079)	This study

Abbreviations: Gm^r^, gentamicin resistance.

### Determination of the host specificity of phages JG004 and PaP1

To determine the phage specificity, top agar plates were prepared by adding 200 µl of *P. aeruginosa* PA1 or PAO1 from an overnight culture to 10 ml of LB agar (0.7%), after which they were plated on LB agar plates. Phages JG004 and PaP1 stock solutions were diluted to 10^−4^. The diluted solution (1 µl; approximately 500 phage particles) was spotted onto the top agar plates. The plates were incubated at 37 °C overnight, and the appearance of the clear lysis zones was examined. Each phage was tested thrice against the bacterial strains PA1 and PAO1.

### Plaque assay and adsorption assay

Phage titers were determined by plaque assay on host strain PA1 or PAO1 in LB medium as previously described [29]. Phage adsorption assays for PAO1 and PA1 were performed as previously described [43]. Briefly, 2 ml late-exponential-phase bacterial cells were mixed with 10 µl of diluted phages (approximately 10^5^ phage particles). After a 5 min incubation at 37 °C with shaking, the mixture was centrifuged and filtered with 0.2 µm filter, and the number of phages in the supernatant was determined by plaque assay. Percent adsorption of the phage was calculated using the equation [(initial titer − residual titer in the supernatant) / initial titer] × 100% as previously described. Error bars represent the standard deviation of the three independent experiments.

### Isolation of spontaneous mutants of *P. aeruginosa* phage JG004

A total of 500 µl of phage JG004 with a titer of approximately 5 * 10^10^ pfu/ml, which was determined by plaque assay, was mixed with 200 µl of *P. aeruginosa* strain PA1 and plated in a double-layer plate. Approximately 50 plaques formed after an overnight culture. Five clear plaques were transferred into the LB medium and serially diluted to 10^−6^. The plaque assay was repeated with 10 µl of each dilution to obtain well-separated plaques. The same procedure was repeated twice to purify the mutant phages.

### DNA isolation and sequencing

Genomic DNA of the mutant phage was extracted as previously described [22]. The whole region encoding the baseplate and tail fiber was amplified by PCR with primers (Table S1). This procedure was performed using Taq polymerase obtained from Promega (Madison, WI, USA) according to the manufacturer’s instructions. The PCR products were then purified and sent to Laragen Inc. (Culver City, CA, USA) for Sanger sequencing.

### Generation and isolation of chimeric PaP1 phages

A fragment encompassing 770 nucleotides upstream and 1761 nucleotides downstream of the coding region of ORF84, which are both 99% identical to the corresponding region of PaP1, was amplified from purified phage JG004 DNA by PCR using the primers 84-u (5′-t G G A T C CCTGGAACAGGGAGTTGGATA) and 84-d (5′-t C T G C A GCTACGGTGTAATAATAGAAGGAGA). Underlined nucleotides indicate recognition sequences for BamHI and PstI, respectively. The PCR fragment was gel purified and ligated into the T vector (pMD®19-T Simple vector, Takara Bio Code: D104A), resulting in plasmid pT-84. Recombinant phage fragment on plasmid pT-84 was cut with BamHI–PstI and ligated into the BamHI–PstI-digested pUCP24, an *Escherichia* *−Pseudomonas* shuttle vector [44], resulting in pUCP-84. Then, pUCP-84 was introduced into the host strain PA1 by electroporation [45].

The obtained transformant strain PA1-p84, which was sensitive to phage PaP1 but not to JG004, was cultivated in LB medium supplemented with 60 µg/ml gentamicin until exponential phase. The strain was then infected with phage PaP1 at a multiplicity of infection of 0.01 and incubated at 37 °C with shaking for 5 h before a complete lysate was obtained. Cell debris was removed by centrifugation and filtration. Supernatant (1 ml) was mixed with a 0.5 ml overnight culture of PAO1. The mixture was added to 15 ml of LB medium containing 0.7% agar, overlaid on LB plates, and then cultured at 37 °C overnight. Five plaques containing putative recombinant PaP1 phages were picked from a total of 50 and purified twice by plaque assay for further analysis.

To further confirm these recombinant PaP1 phages carrying ORF84 from JG004, a set of JG004-specific primers (JG004-84specific-U: TTGGTTACTCCTGATGATAGCTCG and JG004-84specific-D: CAATCTGGCTTCAAAGTCATCATAT) was used to amplify this fragment from a DNA template of five recombinant PaP1 phages, wild-type PaP1, and JG004. To exclude the possibility of contamination by other phages, primers PaP1-U: ATCGCATATCCAACGCCTC and PaP1-D:ATCTTGTCGCCCTACACCG were designed to amplify a PaP1-specific fragment from these mutant phages. The PCR products were sent for sequencing and compared with the sequence of PaP1 or JG004.

## Supporting Information

Figure S1 Adsorption assay of mutant bacteriophages to *P. aeruginosa* PA1 and PAO1. Mutant phages JG004-m0、JG004-m2、JG004-m4、JG004-m6、JG004-m7 bind to both PA1 and PAO1.
(DOCX)

Table S1 Primers for amplification of the tail fiber and baseplate region of JG004-m0 and JG004.
(DOCX)
