# Supplementary material for: Mapping the Tail Fiber as the Receptor Binding Protein Responsible for Differential Host Specificity of Pseudomonas aeruginosa Bacteriophages PaP1 and JG004
Source: PLoS One. 2013 Jul 9;8(7):e68562. doi: 10.1371/journal.pone.0068562 (PMC3706319; doi:10.1371/journal.pone.0068562)

**Figures S1. Adsorption assay of mutant bacteriophages to *P. aeruginosa* PA1 and PAO1.** Mutant phages JG004-m0、JG004-m2、JG004-m4、JG004-m6、JG004-m7 bind to both PA1 and PAO1.


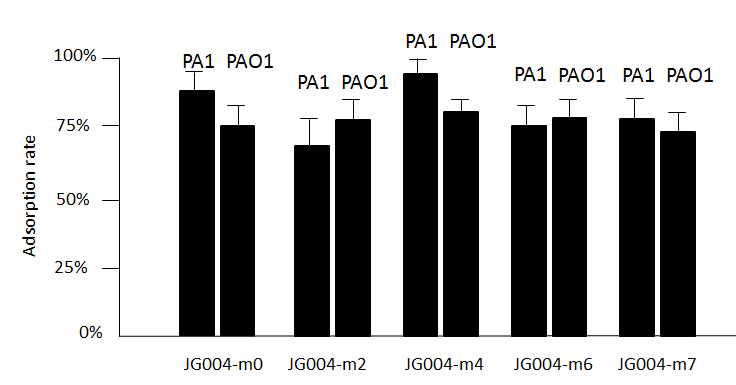

Supplement: Figure S1 — Mutant phages JG004-m0、JG004-m2、JG004-m4、JG004-m6、JG004-m7 bind to both PA1 and PAO1. (DOCX) [file pone.0068562.s001.docx]
