# Supplementary material for: Mapping the Tail Fiber as the Receptor Binding Protein Responsible for Differential Host Specificity of Pseudomonas aeruginosa Bacteriophages PaP1 and JG004
Source: PLoS One. 2013 Jul 9;8(7):e68562. doi: 10.1371/journal.pone.0068562 (PMC3706319; doi:10.1371/journal.pone.0068562)
Supplement: Table S1 — Primers for amplification of the tail fiber and baseplate region of JG004-m0 and JG004. (DOCX) [file pone.0068562.s002.docx]

**Table S1**. Primers for amplification of the tail fiber and baseplate region of JG004-m0 and JG004

| **JG-P1U** | TCTGCACGTAGTCGCCTGTAT |
| --- | --- |
| **JG-P1D** | TCAAATGCCTTCTCAATCCTG |
| **JG-P2U** | AACAGCCGTAAGACTATAAGCA |
| **JG-P2D** | CACTAGGTTGTCCAGAGCCAC |
| **JG-P3U** | TCTGGGTTGTCGGTATCGTTT |
| **JG-P3D** | TCGGCGTAGTTGCTCCATCG |
| **JG-P4U** | CCATGCCTTGAGTCTGTCCT |
| **JG-P4D** | GCTTGGTATCGCTGTTGACG |
| **JG-P5U** | GACAGTCTAGGCTACACTCACAG |
| **JG-P5D** | GATCATCCGATACTCTTCGTC |
| **JG-P6U** | CATAAGCCGTAGAGTCAAACA |
| **JG-P6D** | AACTGGACATTATCGGAGACA |
| **JG-P7U** | CAACCAGAAATACCTGCTCCT |
| **JG-P7D** | GCTTTCGCAGACTTTACCACA |
| **JG-P8U** | AATGCCTCTGTGCGGATTAA |
| **JG-P8D** | GCTGGTAGTTTGACGGGAGA |
| **JG-P9U** | GTAATGACTGATGCCCAAATC |
| **JG-P9D** | CCCTGATACTCTGTGACGCTA |
| **JG-P10U** | AGCAGTGTTAGCCAATCCAG |
| **JG-P10D** | AGAACCCATTCCGACAAGAT |
| **JG-P11U** | CTTGAACATCCAGTGCCTTTC |
| **JG-P11D** | GATACAGCGACTATGCCTTCTT |
